# A dual-consistency semi-supervised learning method for histopathology image segmentation

**DOI:** 10.1186/s12880-026-02281-8

**Published:** 2026-03-24

**Authors:** Mingjian Xie, Weifeng Zhang, Yiqun Geng, Yuting Duan, Dekai Wang, Hongzhong Tang, Liangli Hong

**Affiliations:** 1 College of Automation and Electronic Information, Xiangtan University, Xiangtan, 411105 China; 2 The Second Affiliated Hospital of Fujian Medical University, Quanzhou, 362000 China; 3 Key Laboratory of Molecular Pathology and Personalized Medicine, Center of Collaborative and Creative Center, Department of Pathology, Shantou University Medical College, Shantou, 515041 China; 4 Department of Pathology, The First Affiliated Hospital of Shantou University Medical College, Shantou, 515041 China

**Keywords:** Semi-supervised learning method, Hierarchical consistency, Coarse-fine grained consistency, Histopathology image segmentation

## Abstract

Segmenting tumor regions from the whole-slide images (WSIs) is crucial for quantitative characterizing of the tumor microenvironment and supporting clinical diagnosis. Compared with classification tasks, segmentation requires pixel-level annotations at gigapixel resolution, which makes manual labeling particularly costly. To address the issues, we propose a dual-consistency semi-supervised learning method for histopathological image segmentation, including hierarchical consistency (HC) and coarse-fine grained consistency (CFGC) regularizations. HC enforces prediction alignment across multiple levels in the decoding, encouraging the network to learn coherent representations and improving robustness when training with unlabeled data. Furthermore, CFGC establishes consistency between coarse-grained and fine-grained segmentation results through a multi-scale convolution module (MSC) that enhances feature propagation from the encoder to the decoder. The proposed experimental results demonstrate that the proposed method based on HC and CFGC for semi-supervised histopathology image segmentation can finely segment cancerous regions with little annotation data, assisting pathologists in diagnosis.

## Introduction

Automatic segmentation of tumor tissues from WSIs has become as a crucial yet challenging task in computational pathology [1, 2]. It plays a key role in quantitatively analyzing tumor microenvironments, assessing effects of immune responses and supporting decision-making for disease diagnosis. Moreover, computer-aided diagnosis of WSIs is expected to improve the efficiency and reliability of pathological assessments [3, 4]. Recent progress in deep learning, particularly advancements in convolutional neural networks (CNNs), has created new opportunities for applications in digital pathology, such as cancer classification [5] and segmentation [6], cell detection [7], and mutation prediction [8]. However, deep learning-based methods for automatic histopathology image analysis still face significant challenges, primarily because their performance heavily relies on fully supervised models that require large amounts of labeled data for effective training. The process of obtaining large quantities of fine-grained annotation for WSI analysis is highly time-consuming and labor-intensive due to the gigapixel size and complex morphological features of these images. This impedes the further advancement of traditional supervised algorithms. In recent years, semi-supervised image segmentation, by leveraging limited labeled data and abundant unlabeled data, holds great promise for enhancing model performance and alleviating pathologists’ workload in cancer diagnosis.

Semi-supervised learning (SSL) enables models to learn effectively from limited labeled data by leveraging the complementary strengths of supervised and unsupervised strategies, ultimately producing strong predictive outcomes [9, 10]. Representative approaches in the field of SSL can be broadly classified into two major groups: pseudo-labelling-based methods [11–14] and consistency-based methods [15–19].

Pseudo-labeling follows a simple idea: the model trained on annotated samples infers tentative labels for unannotated inputs, allowing these predictions to act as additional supervision signals. Taking classification tasks as an example, Tolkach et al. [20] used a pseudo-labeling-based, semi-supervised strategy to train the CNN network for Gleason classification. In segmentation tasks, Su et al. [12] developed two subnetworks to identify reliable labels by comparing the confidence scores of their predictions by leveraging a mutual learning strategy. This method can effectively address medical image segmentation with limited labeled data. Seibold et al. [13] presented a different view where a small number of labeled images was used as a reference to match pixels in an unlabeled image to generate pseudo-labels for medical image semantic segmentation on online pseudo-labels in semantic segmentation. The effectiveness of these approaches is strongly bound to the reliability of the initial supervised model, because the model must generate high-confidence predictions before they can be reused as supervision signals.

Recently, consistency-based SSL methods have shown to work well in many natural image applications, including classification and segmentation tasks [9, 10], and have attracted increasing attention. Consistency-based SSL methods are built upon the notion that an unlabeled sample should yield stable predictions even when perturbed in different ways. A representative work, FixMatch [9] constrained the model to maintain consistent outputs for an unlabeled input even when weak and strong augmentations are applied. FlexMatch [10] further employed a curriculum pseudo-labeling strategy that adaptively updates the confidence threshold for each category, enabling the model to produce more reliable pseudo-labels. Consistency-based regularization has become a major focus in semi-supervised learning for medical image analysis. For example, Su et al. [21] proposed a novel global and local consistency loss, and performed the nuclei classification task based on the mean-teacher framework. Wang et al. [22] proposed a consistency-aware pseudo-labeling method combining Vision Transformer (ViT) and CNN to enhance feature learning. Zhou et al. [23] introduced X-Net, a network for biomedical image segmentation, which uses wavelet transformations and consistency regularization to reduce learning biases between low- and high-frequency components. Zhang et al. [24] proposed a consistency-based adaptive pseudo-labeling method that defined a dynamic adaptive threshold based on the current learning status of the model to generate pseudo-labels for better utilization of unlabeled data. Our early work, presented by Xie et al. [25], designed a multi-resolution consistency semi-supervised active learning framework, where images at different resolutions are encouraged to generated similar predictions for histopathology image classification. Luo et al. [26] proposed URPC, a consistency framework for semi-supervised medical image segmentation, which combines pyramid consistency and uncertainty rectification to enforce consistency across multi-scale predictions.

Although these methods have shown promising results in biomedical image classification and segmentation, they have some issues to be addressed. On the one hand, existing consistency-based SSL approaches may suffer a drop in model performance due to their reliance on the mean teacher architecture, which is sensitive to noisy pseudo-labels by the teacher model and lead to inconsistent guidance during training. On the other hand, they may focus on perturbing within input space and model changes, ensuring consistencies only at the final layer of the network in different models for different perturbed images. As result, incorrect guidance information from unlabeled data may be propagated layer by layer in CNN architecture [26], leading to inconsistent representation between low-level and high-level features learned by the model.

Inspired by the pyramid consistency mechanism in URPC, we adopt HC as a foundational constraint to enforce prediction consistency across multiple decoder stages. However, pyramid-level consistency alone does not explicitly model semantic coherence across different granularity levels. To address this limitation, we introduce a MSC to enhance multi-scale feature representation in the decoder, and further incorporate a CFGC mechanism to enforce semantic alignment between coarse and fine segmentation outputs. By jointly integrating hierarchical consistency, multi-scale feature enhancement, and coarse–fine semantic regularization, our framework promotes more coherent cross-scale information propagation during decoding. The main contributions of our method are as follows.

1. We introduce hierarchical consistency between different levels of the decoder to ensure that features learned at various layers remain consistent, which is crucial for building a reliable and robust segmentation model for histopathology images.
2. We present a multi-scale convolution module that captures rich contextual information by integrating high-level semantic features with low-level decoder details for fine-grained WSI tumor segmentation. In addition, we introduce CFGC to enforce consistency between coarse- and fine-grained predictions, further improving the effectiveness of semi-supervised segmentation.
3. We perform extensive experiments on three histopathology datasets and show that our method achieves competitive performance under limited annotations, outperforming mainstream SSL semantic segmentation approaches.

## Related work

### Deep learning-based histopathology image segmentation

Deep learning has been widely applied into histopathology image analysis, particularly classification and segmentation tasks using fully supervised frameworks [5, 6]. Micro-Net [27] was designed to segment heterogeneous cell and tissue components by exploiting multi-scale representations and feature fusion. Transformer-based architectures have further obtained promising cell segmentation performance. For example, the multimodality cell segmentation benchmark presented in [28] demonstrated that Transformer models effectively handle diverse microscopy images across tissue types. Haru-Net [29] adopted a hybrid attention mechanism, combining spatial and channel attention to highlight informative cellular regions, thereby improving nuclei segmentation in histopathology images.

Histopathology image segmentation aims to localize high-level pathological regions such as tumor areas or cancerous structures in WSIs. An adaptive weighting multi-filed-of-view segmentation network [30] was introduced to learn contextual features from multiple networks for segmenting cancerous regions. An efficient follicular segmentation method was proposed for thyroid cell WSIs [31], which filters irrelevant regions via a classification branch and integrates fine-scale features into the spatial pyramid pooling module to boost segmentation accuracy. MAMC-Net [32] integrated multi-scale feature learning to handle the large resolution and structural variability of WSIs, achieving improved tumor segmentation performance. Despite these recent developments, most existing supervised segmentation approaches rely heavily on fine-grained pixel-level annotations provided by expert pathologists. However, generating such dense labels for large-scale WSIs is labor-intensive, time-consuming, and often impractical. Consequently, the limited availability of annotated data restricts the applicability of fully supervised deep learning models in computational pathology.

### SSL-based biomedical image segmentation

Because pixel-level annotations in histopathology image segmentation are expensive, many studies have explored semi-supervised learning (SSL) to leverage unlabeled data. A central challenge in SSL lies in obtaining reliable supervision signals for unlabeled samples. Pseudo-labeling is a widely adopted strategy, where a teacher model generates pseudo-labels to supervise a student model. For example, Shaw et al. [33] employed a teacher–student framework in which the teacher produces pseudo-labels for unlabeled data that are subsequently used to train the student model. Basak et al. [34] further exploited pseudo-labels to guide contrastive learning, improving multi-class segmentation performance on gland and tumor segmentation tasks. Despite their promising performance, pseudo-labeling-based methods are sensitive to noisy predictions and typically rely on confidence thresholds to select reliable pseudo-labels, which may limit their effectiveness under extremely limited annotations. To alleviate this issue, recent work such as PICK [35] integrates pseudo-label guidance with masked image modeling, enhancing robustness and utilization of unlabeled data. Beyond segmentation, Zeng et al. [36] proposed PEFAT, a semi-supervised medical image classification method that models pseudo-label reliability via loss distribution estimation and feature-level adversarial training to better exploit low-confidence unlabeled samples. Nevertheless, these approaches still depend on pseudo-label assignment and threshold-based selection, motivating alternative SSL strategies that reduce reliance on explicit pseudo-label supervision.

Consistency regularization constitutes another major SSL paradigm, which encourages invariant predictions under different perturbations and has demonstrated strong performance in biomedical image segmentation. For instance, Zhou et al. [37] proposed a mean-teacher framework that enforces consistency regularization through template-guided and perturbation-aware sample selection to improve supervision on unlabeled data. However, most existing consistency-based approaches primarily focus on reducing prediction variance induced by input-space perturbations [9, 10, 15–19, 24, 25, 37]. Although more recent studies have extended consistency regularization to model perturbations [22] and feature-level perturbations [23], the hierarchical nature of CNN representations and cross-scale relationships remains insufficiently explored.

In addition to standard mean-teacher consistency, several recent studies have explored alternative supervision signals for semi-supervised medical image segmentation. Zeng et al. [38] proposed an uncertainty co-estimator framework with dual mean-teacher modules to collaboratively estimate multi-source uncertainty and enforce both internal and cross-module consistency, thereby improving robustness on unlabeled data. Du et al. [39] presented a co-training-based semi-supervised approach that integrates dual diversity learning with pseudo-label correction, where data- and feature-level diversity encourages complementary learning while inconsistency-aware correction suppresses noisy pseudo-labels under limited annotations. Subsequently, Du et al. [40] proposed a semi-supervised segmentation framework that combines diversity feature fusion with dual consistency learning to enhance multi-scale medical image segmentation. Similarly, LeFeD [41] introduced a discrepancy-driven semi-supervised segmentation framework that leverages feature-level inconsistencies between dual decoders with prediction-level consistency regularization to enhance representation learning under limited annotations.

Despite these advances, most prior SSL approaches are still built upon the mean-teacher paradigm and rely on enforcing prediction consistency between teacher and student models. Although these approaches have achieved promising results, they primarily focus on output-level invariance under perturbations and insufficiently explore the hierarchical feature propagation inherent in convolutional neural networks. Specifically, the relationships between low-level detailed features and high-level semantic representations across decoding stages are often underutilized [23, 26, 36]. Moreover, limited attention has been paid to maintaining structural coherence between coarse-grained and fine-grained segmentation outputs during the transition from encoding to decoding, which is crucial for accurate multi-scale histopathology segmentation.

Recently, unified semi-supervised paradigms have emerged to learn task-agnostic representations across multiple datasets or modalities. TextMoE [42] introduced a text-enhanced mixture-of-experts framework that incorporates textual priors to jointly learn from heterogeneous CT–text and X-ray–text data within a unified semi-supervised segmentation model. VerSemi [43] presented a versatile semi-supervised segmentation paradigm that unifies multiple tasks through dynamic task prompting and synthetic-task consistency, enabling scalable learning from unlabeled data. These approaches focus on unification and scalability by incorporating external semantic priors. In contrast, our work focuses on hierarchical and cross-scale consistency modeling within a vision-only framework for histopathology image segmentation, which is particularly suitable for datasets where structured textual descriptions are often unavailable.

To this end, we propose a dual-consistency semi-supervised framework for histopathology image segmentation. Specifically, we enforce hierarchical consistency (HC) across multiple decoding stages to encourage coherent feature representations under limited supervision. Furthermore, inspired by prior multi-scale segmentation studies [32], we introduce a coarse–fine grained consistency (CFGC) regularization through a multi-scale convolutional module that bridges low-level detailed features and high-level semantic information. By enforcing consistency between coarse-grained and fine-grained segmentation outputs, the proposed framework reduces discrepancies during the transition from encoding to decoding, leading to more accurate and robust tumor region segmentation with minimal annotation data.

## Methods

### Network architecture

**Fig. 1:**
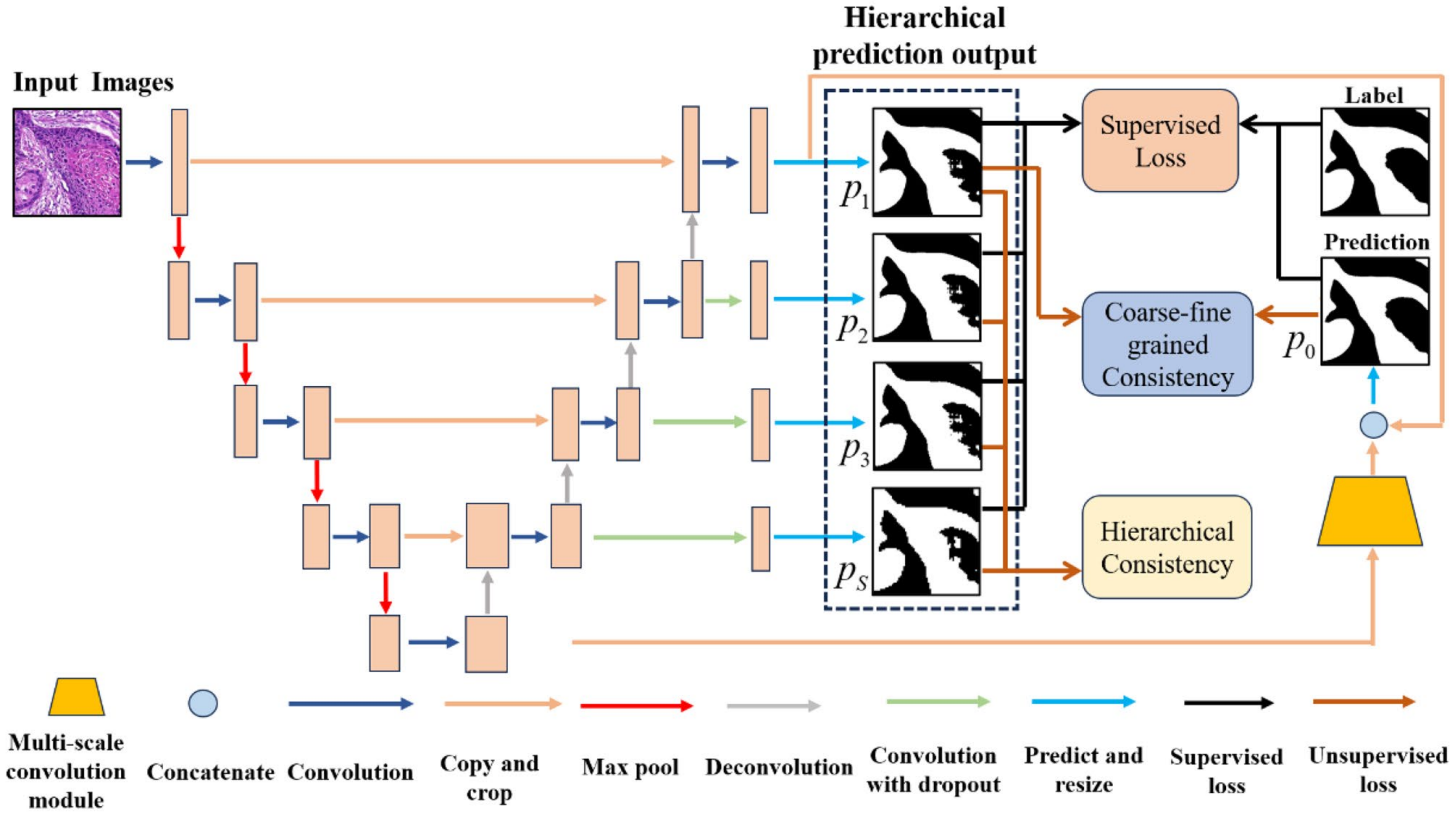
Illustration of the proposed network

In this study, we propose a dual-consistency semi-supervised learning framework for histopathology image segmentation, as illustrated in Fig. 1. The method is built upon a U-Net backbone including an encoder–decoder architecture. The encoder consists of four convolutional blocks followed by max-pooling operations, whereas the decoder is composed of four convolutional blocks and corresponding de-convolution layers. Skip connections are employed to transmit spatial features from the encoder to the decoder, ensuring the recovery of structural information lost during down-sampling. Unlike the classic U-Net architecture [44], our decoder incorporates convolutional layers with dropout at multiple stages to generate multi-level predictions. These intermediate predictions are further up-sampled to be consistent, scale-aware outputs. In addition, a multi-scale convolution module is integrated into the decoder to enhance the interaction between shallow and deep feature representations. This design facilitates the recovery of fine-grained structure details, especially for small or sparsely distributed tissue regions. By effectively fusing multi-scale features, the proposed framework is capable of producing fine-grained and robust segmentation results for histopathology images, even under limited supervision.

### Multi-scale convolution module

**Fig. 2:**
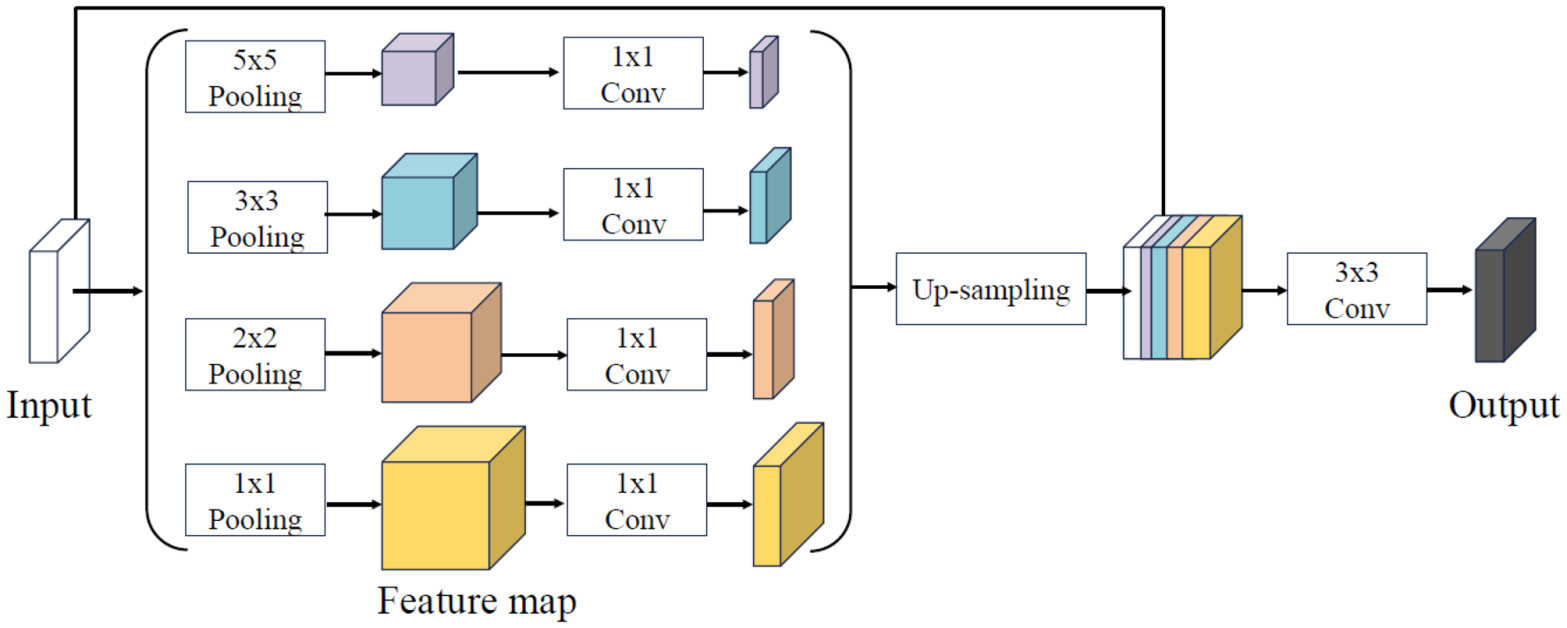
Structure of the multi-scale convolution module

We design a multi-scale convolution module (MSC), as illustrated in Fig. 2. This module applies four pooling operations with kernel sizes of 1 × 1, 2 × 2, 3 × 3, and 5 × 5 to perform multi-scale down-sampling. The smaller pooling kernels preserve fine-grained structural details, whereas the larger kernels capture broader contextual information [34]. The resulting feature maps are then up-sampled to a uniform spatial resolution and concatenated, followed by a 3 × 3 convolutional layer to generate the effective representation. This module effectively integrates features across different receptive fields, thereby enriching contextual information and enhancing the recovery of subtle image details.

### Dual-consistency regularization

#### Hierarchical consistency regularization

In a semi-supervised learning (SSL) segmentation, the training set consists of both annotated and unannotated samples. We denote the annotated set as ${D_l}=\{ ({x_i},{y_i})\} _{{i=1}}^{{{N_l}}}$, where ${x_i}$ is an image patch and ${y_i}$ is its corresponding segmentation mask. The unannotated set is defined as ${D_u}=\{ ({u_i})\} _{{i=1}}^{{{N_u}}}$, containing only image patches without ground-truth. Here, ${N_l}$ and ${N_u}$ refer to the number of annotated and unannotated samples, respectively. It should be highlighted that the unannotated dataset is substantially larger than the annotated dataset, with ${N_l} \ll {N_u}$.

We denote the multi-level segmentation outputs in Fig. 1 as $[{p_1},{p_2},{p_3},...,{p_S}]$, where *S* represents the total number of prediction levels. Among them,${p_1}$ represents the coarse-grained segmentation result, which is the prediction at the shallowest level of the network, wheras ${p_S}$ denotes the segmentation result at the deepest level of the segmentation network, ${p_s}=softmax\left({convolution\left({Up\left({{D_{s+1}}\left(x \right)} \right)} \right)} \right){\text{ }}$, where ${D_{s+1}}\left(x \right)$ denotes the feature representation of the $s+1$-th decoder stage, $Up\left(\cdot \right)$ denotes an upsampling operator that restores the spatial resolution to match the input image, and $softmax\left(\cdot \right)$is applied over the channel dimension to generate normalized probability maps. For consistency of notation, the segmentation result obtained from the fused multi-scale features is referred to as the fine segmentation result, and is denoted by ${p_0}$.

For annotated data, the supervised loss that constrains the multi-level segmentation predictions with respect to the ground-truth labels can be defined as

1 $${L_{sup}}=\sum\limits_{{s=0}}^{S} {{\alpha _s}L({p_s},y)} $$

where$L(\cdot)$is the combination of Dice loss and cross-entropy loss, ${\alpha _s}$ is the weighting factor for different scale s, y is the ground-truth. Following the setting in URPC [26], we fix all 𝛼_𝑠_ to 1.0, assigning equal supervision weight to each multi-level prediction. Combing Fig. 1 and Eq. (1), it can be seen that the model learns representations across multiple decoder levels. The deeper layers contribute to extracting more discriminative semantic features, whereas the shallower layers provide complementary spatial and positional information.

For unannotated samples, we introduce hierarchical consistency between the multi-level outputs to encourage the model to produce similar predictions at different levels for histopathological images. To reduce computational complexity, the multi-level prediction results are summed and averaged to obtain the overall result. Thus, the hierarchical consistency across multi-level results can be transformed into the consistency between the result of each level and the average result. It can be expressed using the L_2_ distance as follows:

2 $${p_{avg}}=\frac{1}{S}\sum\limits_{{s=1}}^{S} {{p_s}} $$

3 $${L_{hc}}=\frac{1}{S}\sum\limits_{{s=1}}^{S} {||{p_s} - {p_{avg}}|{|_2}} $$

It can be observed that this consistency can reduce the diversity of prediction results at different levels and enhance the robustness of the model.

To generate multi-level predictions, up-sampling operations are applied to align them with the input size. However, this process may incur noises and boundary distortions, increasing the prediction uncertainty of the model. Luo et al. [26] proposed an uncertainty estimation method based on multi-level predictions. In this approach, the differences between multi-level prediction results are computed in a single forward pass, with larger discrepancies indicating higher uncertainty. Therefore, we utilize the uncertainty estimation to adjust the loss function of the hierarchical consistency, assigning higher weights to the reliably predicted regions while ignoring the unreliable predictions. The modified hierarchical consistency loss function is defined as follows:

4 $${L_{urc}}=\frac{1}{S}\sum\limits_{{s=1}}^{S} {\frac{{\sum\nolimits_{i} {(||p_{s}^{i} - p_{{avg}}^{i}|{|_2} \cdot w_{s}^{i})} }}{{\sum\nolimits_{i} {w_{s}^{i}} }}} $$

5 $$w_{s}^{i}={e^{ - D_{s}^{i}}}$$

6 $$D_{s}^{i}=\sum\nolimits_{i} {KL(p_{s}^{i},p_{{avg}}^{i})} $$

where $KL(\cdot)$ represents Kullback-Leibler (KL) divergence, i denotes the i-th pixel. ${D_s}$ represents the uncertainty map of the prediction${p_s}$, and ${w_s}$ represents the corresponding uncertainty weight. From Eq. (6), a larger ${D_s}$ is indicative of high inconsistency between ${p_s}$ and ${p_{avg}}$, and higher uncertainty in the model prediction. Thus, ${w_s}$ is assigned a lower weight in Eq. (4), generating more reliable and robust training.

#### Coarse-fine grained consistency regularization

In the classic U-Net segmentation network, skip connections fuse features from the encoder and decoder. However, interactions among different levels within the decoder are relatively limited, which constrains the effectiveness of feature fusion. To address this limitation, we introduce a multi-scale convolution module that facilitates feature integration between the shallow and deep layers of the decoder. In this module, multi-scale representations extracted from the deeper decoder layers are aggregated to compensate for the loss of information induced by repeated up-sampling, thereby enabling the generation of more detailed and fine-grained segmentation results.

We assume that fine-grained segmentation results exhibit higher confidence and can therefore serve as reliable pseudo-labels for unsupervised training. Building on this assumption, we introduce the CFGC, which aims to reduce the discrepancies between coarse-grained and fine-grained predictions and thereby boosting the information transfer from the encoder to the decoder when training with unlabeled data. It can be formulated as follows:

7 $${L_{cr}}=\;{\kern 1pt} \parallel {p_0} - {p_1}{\parallel _{\,2}}$$

where ${p_0}$ and ${p_1}$ are the fine-grained and coarse-grained prediction results, respectively. This consistency allows the model to better leverage the intrinsic features of histopathology images and offers substantial informational guidance for unsupervised training on unannotated data. Thus, the overall unsupervised loss can be defined as follows:

8 $${L_{unsup}}=\beta {L_{urc}}+(1 - \beta){L_{cr}}$$

Here, $\beta$ represents the weighting parameter used to balance HC and CFGC. In this paper, $\beta$ is set to 0.5.

Finally, the supervised loss and unsupervised loss are combined to obtain the overall loss function, which can be expressed as follows:

9 $${L_{total}}={L_{sup}}+\lambda {L_{unsup}}$$

where $\lambda$ is a widely-used time-dependent Gaussian warming up function [45]. $\lambda$ can balance the influence of the supervised loss and unsupervised loss, and is defined as:

10 $$\lambda =k \cdot {e^{\left({ - 5{{\left({1 - t/T} \right)}^2}} \right)}}$$

where k is set to 0.1, *t* represents the current training iteration, and 𝑇 denotes the total number of training iterations, as introduced in [45].

## Experimental results and analysis

### Implementation and parameter settings

All experiments are conducted on a single GeForce GTX 3090 24GB GPU using PyTorch framework. We used SGD with momentum to train our model, the momentum is set to 0.9 and the weight decay is set to 1 × 10^− 4^. The number of epochs was set to 100 and batch size was set to 20. We used a labeled dataset comprising 30% and 20% of the entire training set, respectively, with a 4:1 ratio of unlabeled to labeled samples in each batch.

### Datasets

To validate the effectiveness of the proposed method, we conducted extensive experiments on three different histopathology image datasets, including a self-created dataset and two public datasets, for histopathology image segmentation. They are described as follows:

#### 1st HSUMC dataset

The dataset used in this study was provided by the Department of Histopathology at the First Affiliated Hospital of Shantou University Medical College (1st HSUMC). It consists of 16 hematoxylin–eosin (HE) stained whole-slide images (WSIs) of non-small cell lung cancer (NSCLC), including 10 cases of lung squamous cell carcinoma (LUSC) and 6 cases of lung adenocarcinoma (LUAD). Each slide contains both tumor and non-tumor tissue regions. All WSIs were digitized at 40× magnification using a high-resolution scanner, producing MRXS images with dimensions of approximately 18,000 × 37,000 pixels. Tumor boundaries were delineated independently by two experienced pathologists using the ASAP annotation tool. For the experimental setup, 11 WSIs (7 LUSC and 4 LUAD) were assigned to the training set, while the remaining 5 WSIs (3 LUSC and 2 LUAD) were used for testing.

Preprocessing was performed as follows. Each WSI was divided into non-overlapping image patches at 10× magnification, with a patch size of 512 × 512 and a stride of 256 pixels. Patches exhibiting excessive blur, staining artifacts, contamination, or containing more than 50% background were excluded. A patch was labeled as a tumor sample if the proportion of tumor pixels exceeded 25%, whereas patches composed entirely of non-tumor tissue were categorized as non-tumor.

#### Camelyon17 dataset

The Camelyon17 dataset originated from the Camelyon17 Challenge [46] and was collected by five medical centers in the Netherlands. The dataset contains 210 H&E-stained breast cancer WSIs. The WSIs are provided in TIFF image format, with 110 containing cancerous regions and 100 considered normal slides. Each WSI is manually annotated by histopathology experts. We extracted image patches at the highest resolution, with each patch resized to 256 × 256 pixels, then selected 6000 images for experimentation. Among these, the number of images with cancer area proportions of 0–25%, 25–50%, 50–75%, and 75–100% were 2788, 1219, 906, and 1087, respectively. 

#### BCSS-WSSS dataset

Han et al. published a breast cancer semantic segmentation dataset containing 151 representative ROIs from images of 151 H&E-stained breast cancer sections, each selected by a pathologist [47]. The dataset consists of five different tissue categories: tumor, stroma, lymphocyte infiltration, necrotic, and others, with a total of 8404 images with fine labels. The image size is 224 × 224 pixels, and each image contains one or more tissue categories.

### Evaluation metrics

We employ several quantitative metrics to assess the performance of the segmentation models, including intersection over union (IoU), Dice similarity coefficient (DSC), accuracy, precision, and recall. Among these, IoU and DSC are commonly used to evaluate the degree of overlap between the predicted segmentation and the ground-truth annotation, and are defined as follows:

11 $$IoU=\frac{{|{g_i} \cap {p_i}|}}{{|{g_i} \cup {p_i}|}}$$

12 $$DSC=\frac{{2|{g_i} \cap {p_i}|}}{{|{g_i}|+|{p_i}|}}$$

where ${g_i}$ and ${p_i}$ denotes the *i*-th pixel of the ground truth and the corresponding prediction, respectively. $|{g_i} \cap {p_i}|$ denotes the intersection of the ground truth and the prediction, $|{g_i} \cup {p_i}|$ is the union of the ground truth and the prediction, and $|{g_i}|+|{p_i}|$ is the total of the ground truth and the prediction. It should be noted that both IoU and DSC range from 0 to 1, with higher values reflecting better segmentation performance. Accuracy, precision, and recall are defined as follows:

13 $$Accuracy=\frac{{TP+TN}}{{TP+TN+FP+FN}}$$

14 $$Precision=\frac{{TP}}{{TP+FP}}$$

15 $$Recall{\mathrm{=}}\frac{{TP}}{{TP+FN}}$$

Here,$TP$, $FP$, *FN* and *TN* denote true positive, false positive, false negative and true negative, respectively. Accuracy quantifies the proportion of correct predictions among all predictions, with higher values indicating better overall performance. Precision measures the proportion of true positive predictions among all instances predicted as positive, with higher values indicating fewer false positives.

Recall represents the proportion of actual positive samples that are correctly identified by the model, serving as a measure of the classifier’s ability to detect all positive instances.

### Experimental results and analysis

In order to verify the effectiveness of the proposed method, we compared it with the mainstream semi-supervised semantic segmentation methods, including MT [45], EM [48], UAMT [49], CCT [50], CPS [51], and URPC [26].

#### Comparison using the self-created dataset

Table 1 reports the segmentation performance of all comparative methods on the 1st HSUMC dataset. Under the 30% labeled setting, our proposed method outperformed all other approaches across all evaluation metrics, including IoU, DSC, accuracy, precision, and recall, demonstrating consistently superior performance. Specifically, compared with the MT method, our proposed method achieves improvements of 2.12% and 1.58% in IoU and DSC, respectively, suggesting that it effectively captures richer multi-level features from the decoder and overcomes the limitations of single-level representations. Notably, our method also yielded higher recall values than other methods, with gains ranging from 0.38% to 2.13%, indicating its capability to accurately detect cancerous regions—a critical factor for clinical diagnosis and treatment planning. When compared with URPC, our approach achieves higher IoU, DSC, accuracy, and recall. This improvement can be attributed to two main components: the multi-scale convolution module, which captures semantic features across multiple scales from the deeper decoder layers, and the CFGC strategy, which offers enhanced guidance when learning from unlabeled data. In addition, we further evaluate the proposed method under a more challenging semi-supervised setting with only 20% labeled data. The proposed method achieves an IoU of 0.6978 and a DSC of 0.8041, outperforming most comparison methods. These results further demonstrate the robustness of the proposed framework under limited annotation conditions. The consistent improvements indicate that the method can effectively leverage unlabeled data and maintain strong performance in semi-supervised learning scenarios.

**Table 1:** Comparative results on the 1st HSUMC dataset with 30% and 20% annotated ratio

Method	Scans used	IoU	DSC	Accuracy	Precision	Recall
MT	30%	0.7335	0.8415	0.8778	0.8535	0.8307
EM		0.7285	0.8378	0.8759	0.8520	0.8253
UAMT		0.7300	0.8389	0.8762	0.8538	0.8259
CCT		0.7315	0.8401	0.8773	0.8539	0.8280
CPS		0.7359	0.8432	0.8789	0.8540	0.8336
URPC		0.7427	0.8485	0.8809	0.8547	0.8428
The proposed method		**0.7497**	**0.8536**	**0.8844**	**0.8613**	**0.8466**
MT	20%	0.6942	0.7977	0.8744	0.8227	0.7715
EM		0.6941	0.8017	0.8765	0.8170	0.7828
UAMT		0.6924	0.7965	0.8760	0.8341	0.7622
CCT		0.6918	0.8001	0.8742	0.8113	0.7858
CPS		0.6806	0.7912	0.8743	0.8137	0.7659
URPC		0.6929	0.7971	0.8569	**0.8346**	0.7598
The proposed method		**0.6978**	**0.8041**	**0.8781**	0.8030	**0.8006**

**Fig. 3:**
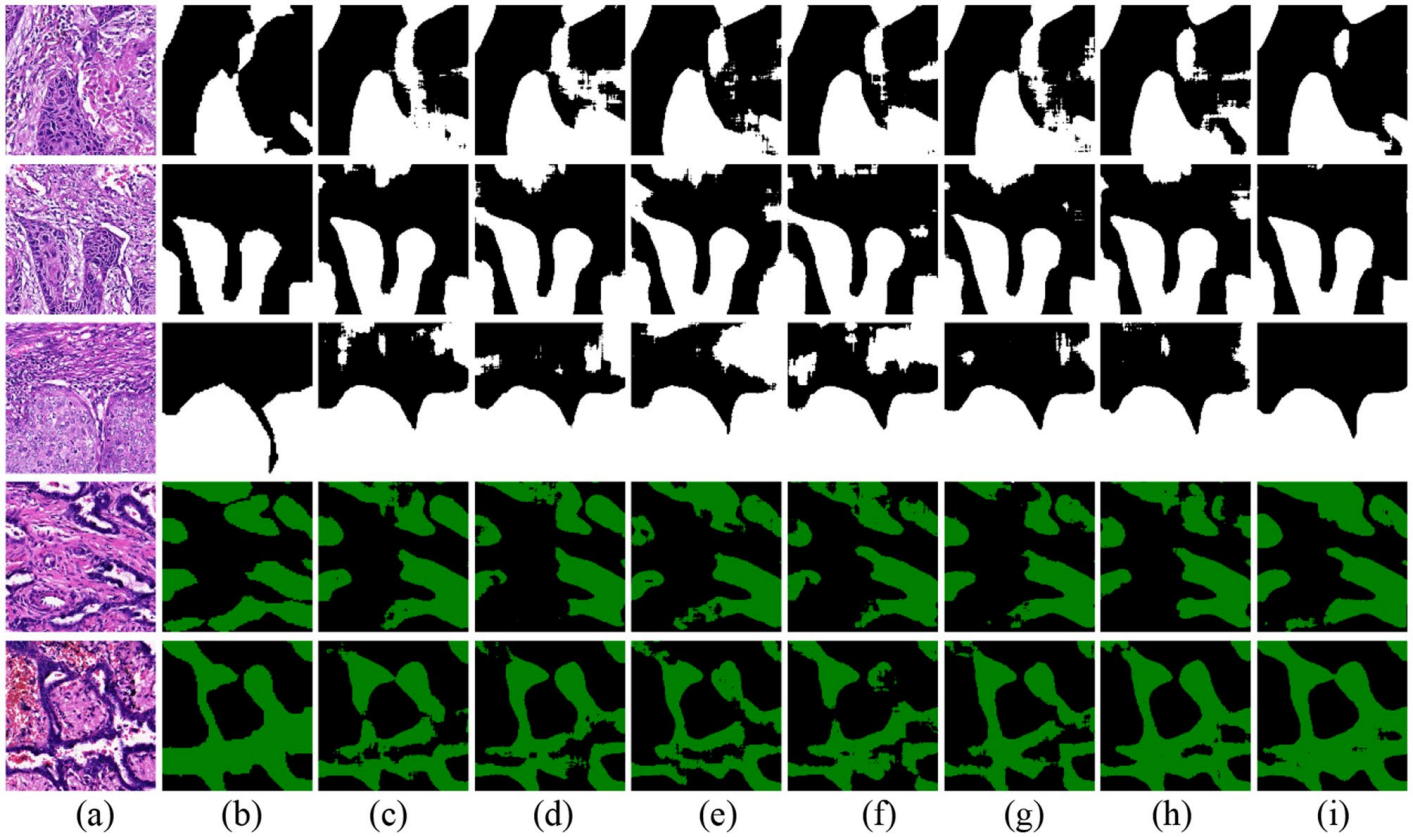
Visualization results of the 1st HSUMC dataset. (**a**) Image. (**b**) Ground truth. (**c**) MT. (**d**) EM. (**e**) UAMT. (**f**) CCT. (**g**) CPS. (**h**) URPC. (**i**) Our method

Figure 3 shows the segmentation results on the 1st HSUMC dataset. In Fig. 3, white represents LUSC, green represents LUAD, and black represents non-tumor regions. The first three rows are LUSC samples with squamous cell structures. As shown in the first and third rows, the MT, EM, UAMT, CCT, CPS, and URPC methods exhibit numerous segmentation errors, particularly misclassifying non-tumor regions as tumor areas.

The last two rows depict LUAD samples with glandular lumen structure and more dispersed cancer regions, increasing the difficulty of accurate segmentation. Most comparative methods show varying degrees of detail loss and often fail to correctly identify cancerous cavities, leading to inaccurate estimates of tumor size and infiltration. Although URPC demonstrates relatively better performance among these methods, the segmentation boundaries remain imprecise. In contrast, our proposed method produces finer segmentation results, effectively preserving the contour and edge features of tumor regions, with higher agreement to the ground-truth annotations.

**Fig. 4:**
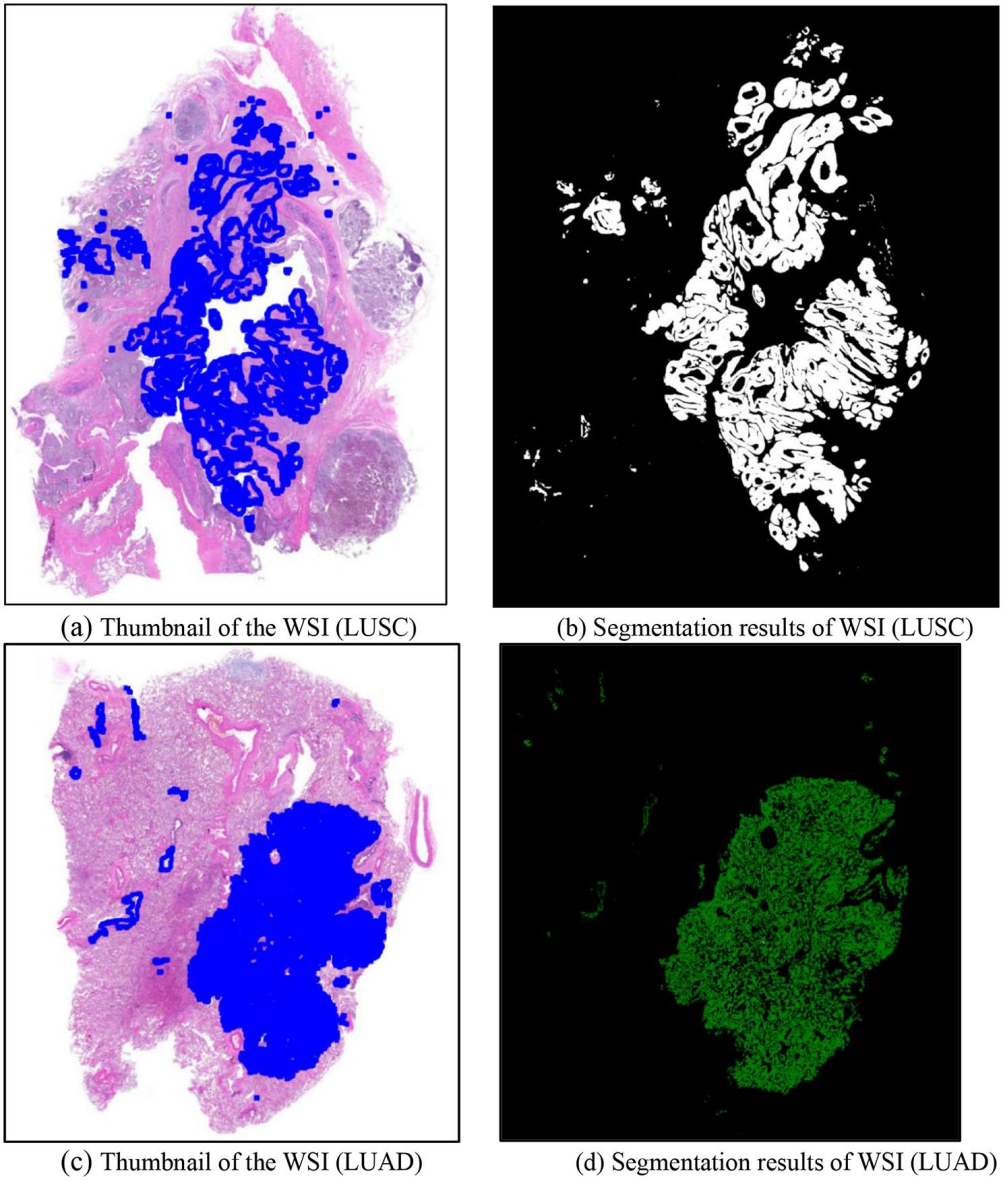
Visualization results of the WSI from the 1st HSUMC dataset. (**a**) Thumbnail of the WSI, (LUSC), (**b**) Segmentation results of WSI, (LUSC), (**c**) Thumbnail of the WSI, (LUAD), (**d**) Segmentation results of WSI, (LUAD)

To demonstrate the segmentation performance of our proposed method on whole-slide images (WSIs), we reconstructed the WSI segmentation by stitching together the results of individual image patches for visualization. Figure 4 presents the WSI thumbnails alongside the corresponding segmentation results, with cancerous regions outlined in blue. The first row shows LUSC samples, while the second row shows LUAD samples. As illustrated, our method achieves accurate and consistent segmentation across the WSIs, exhibiting high visual agreement with the ground-truth annotations.

#### Comparison using the Camelyon17 dataset

To evaluate the generalization ability of the proposed method, we conducted experiments on the Camelyon17 dataset. Table 2 summarizes the overall quantitative results. Under the 30% labeled setting, our method achieves the best performance in terms of IoU, DSC, accuracy, and recall, with respective values of 0.8762, 0.9339, 0.9361, and 0.9392, while maintaining comparably high precision. These results highlight the effectiveness of the proposed approach and demonstrate the benefits of dual-consistency regularization in semi-supervised learning. Compared with the MT method, our proposed approach improves IoU and DSC by 4.04% and 2.35%, respectively, indicating that it can recognize cancer regions more accurately. Under the more challenging 20% labeled setting, our method still achieves strong performance, with an IoU of 0.8550 and a DSC of 0.9213, surpassing URPC by 3.12% in IoU and 1.88% in DSC. Notably, the performance gap becomes more evident under lower labeling ratios, suggesting that the proposed framework is particularly effective in data-scarce scenarios.

**Table 2:** Comparative results on the Camelyon17 dataset with 30% and 20% annotated ratio

Method	Scans used	IoU	DSC	Accuracy	Precision	Recall
MT	30%	0.8358	0.9104	0.9122	0.9067	0.9235
EM		0.8500	0.9188	0.9207	0.9144	0.9297
UAMT		0.8408	0.9134	0.9153	0.9093	0.9254
CCT		0.8403	0.9130	0.9159	0.9092	0.9186
CPS		0.8651	0.9275	0.9300	0.9241	0.9320
URPC		0.8756	0.9335	0.9359	**0.9303**	0.9379
The proposed method		**0.8762**	**0.9339**	**0.9361**	0.9301	**0.9392**
MT	20%	0.7931	0.8839	0.9119	0.9167	0.8560
EM		0.8236	0.9026	0.9256	0.9249	0.8834
UAMT		0.8137	0.8966	0.9216	0.9260	0.8710
CCT		0.7653	0.8661	0.9004	0.9264	0.8169
CPS		0.8248	0.9034	0.9259	0.9225	0.8872
URPC		0.8238	0.9025	0.9258	0.9260	0.8823
The proposed method		**0.8550**	**0.9213**	**0.9391**	**0.9299**	**0.9142**

**Fig. 5:**
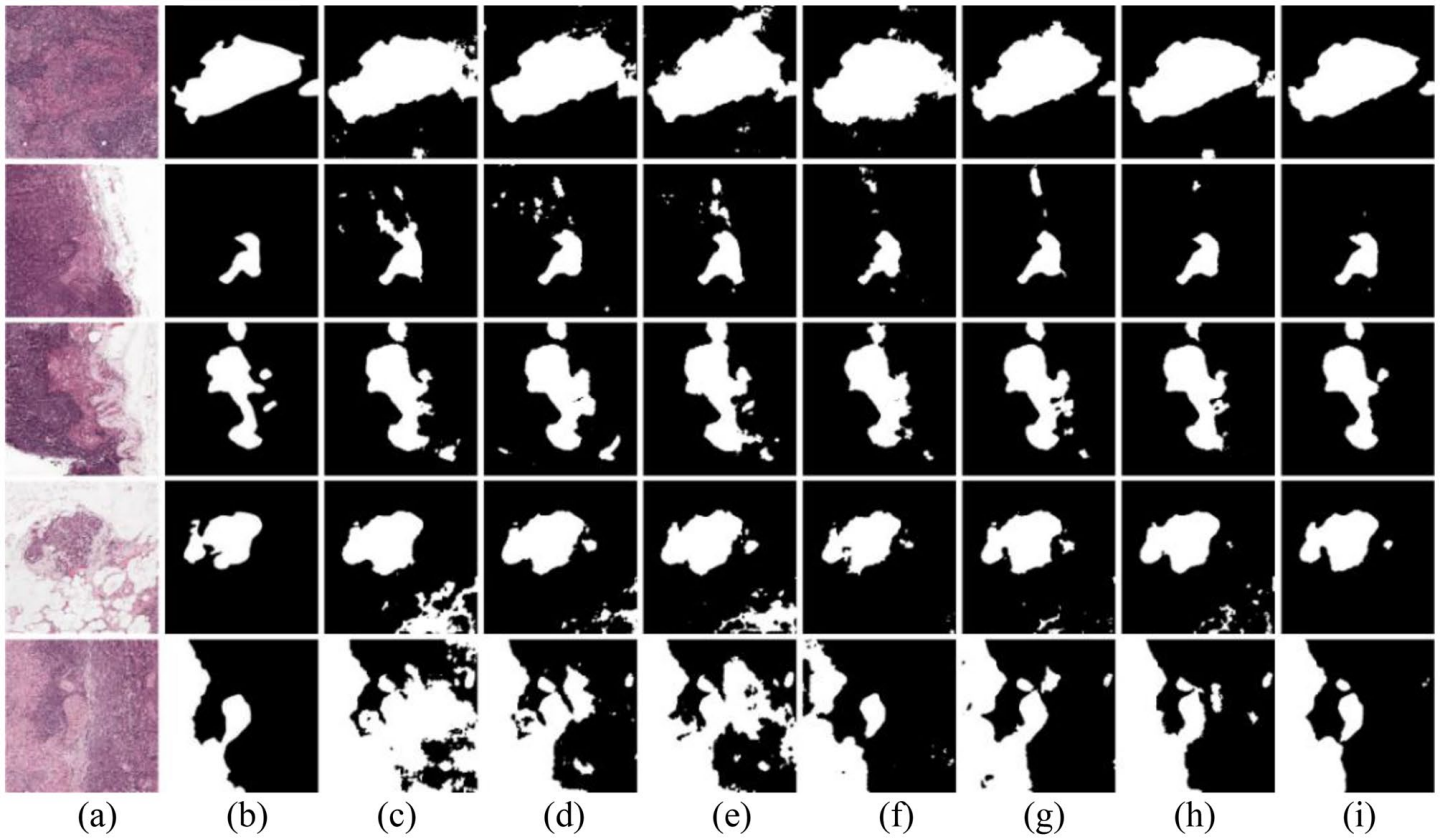
Visualization results of the Camelyon17 dataset. (**a**) Image. (**b**) Ground truth, (**c**) MT, (**d**) EM. (**e**) UAMT, (**f**) CCT, (**g**) CPS, (**h**) URPC, (**i**) Our method

Figure 5 presents the visualization results on the Camelyon17 dataset. White areas represent cancerous regions, while black represents non-tumor areas. The MT, EM, and UAMT methods exhibit substantial mis-segmentation, with non-tumor regions incorrectly classified as tumor tissue. Meanwhile, CPS and URPC generate numerous fragmented regions, leading to inconsistent segmentation results. In addition, CPS require the training of two models simultaneously, leading to higher computation. Compared to other methods, our proposed method shows smoother segmentation boundaries, preserves more complete boundary information of cancerous regions, and presents segmentation results in higher agreement with the ground truth.

#### Comparison using the BCSS-WSSS dataset

We further evaluated the effectiveness of the proposed method on the BCSS-WSSS dataset. Table 3 presents the quantitative segmentation results. This dataset is a multi-class dataset with more complex and diverse cancer characteristics, which makes the segmentation task more challenging. Under the 30% labeled setting, our proposed method achieves the best performance across all evaluation metrics, including IoU, DSC, accuracy, precision, and recall. In particular, our method achieves the largest improvements in IoU and precision, surpassing the competing methods by up to 2.78% and 3.73%, respectively. These results further demonstrate the strong generalization ability and effectiveness of the proposed framework for histopathology image segmentation. Under the more challenging 20% labeled setting, our method still achieves the best performance among all compared approaches, with an IoU of 0.6504 and a DSC of 0.7819, further validating the robustness of the proposed framework under limited annotation conditions.

**Table 3:** Comparative results on the BCSS-WSSS dataset

Method	Scans used	IoU	DSC	Accuracy	Precision	Recall
MT	30%	0.6379	0.7721	0.8264	0.7839	0.7628
EM		0.6438	0.7698	0.8238	0.7767	0.7661
UAMT		0.6365	0.7711	0.8254	0.7829	0.7627
CCT		0.6375	0.7723	0.8236	0.7707	0.7754
CPS		0.6431	0.7765	0.8281	0.7864	0.7684
URPC		0.6480	0.7808	0.8291	0.7899	0.7742
The proposed method		**0.6643**	**0.7926**	**0.8368**	**0.8080**	**0.7832**
MT	20%	0.6158	0.7513	0.8245	0.7753	0.7361
EM		0.6158	0.7515	0.8251	0.7968	0.7270
UAMT		0.6063	0.7441	0.8205	0.7631	0.7338
CCT		0.6139	0.7546	0.8166	0.7771	0.7433
CPS		0.6328	0.7648	0.8304	0.7926	0.7474
URPC		0.6438	0.7790	0.8312	0.7963	**0.7639**
The proposed method		**0.6504**	**0.7819**	**0.8325**	**0.8143**	0.7585

**Fig. 6:**
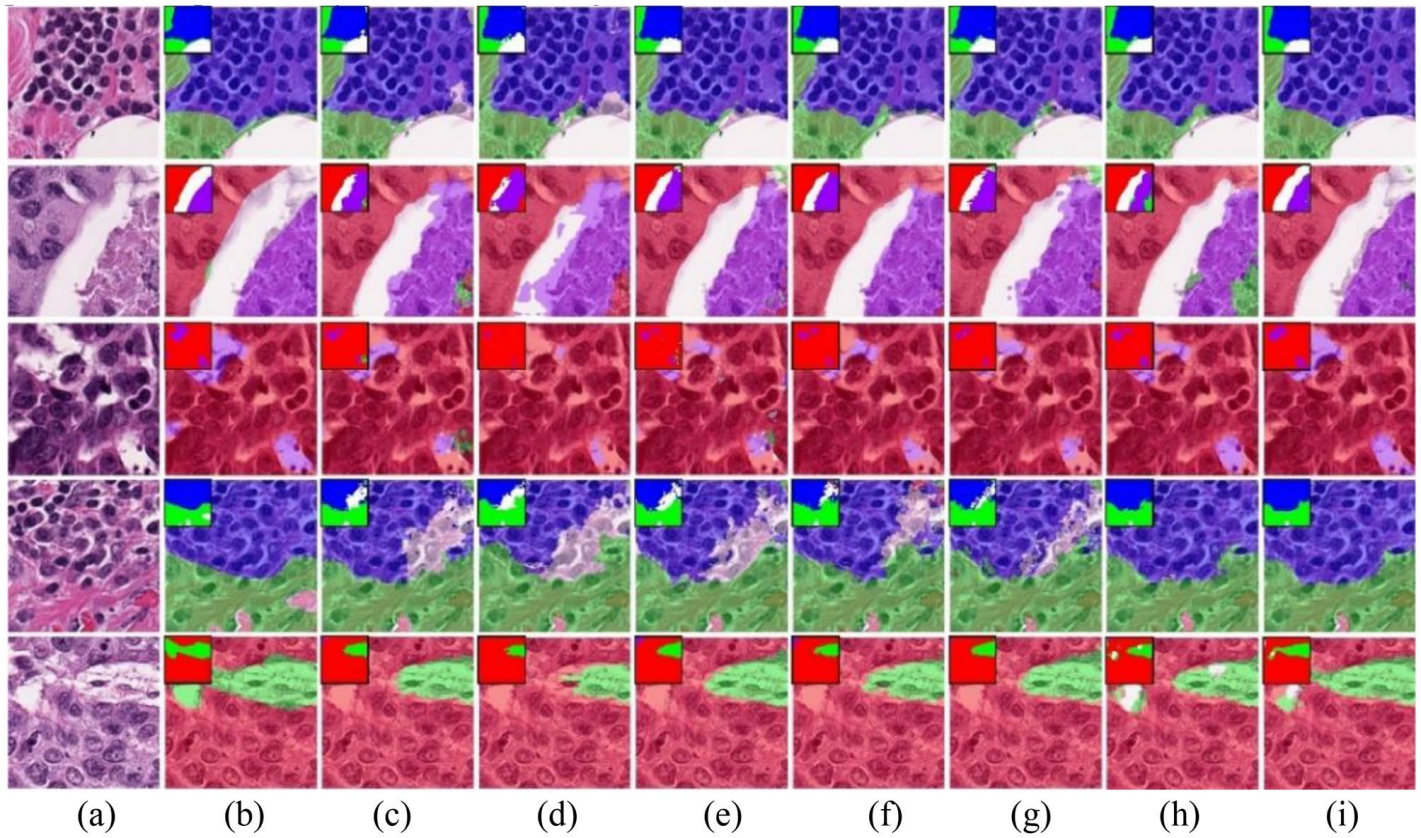
Visualization results of the BCSS-WSSS dataset. (**a**) Image. (**b**) Ground truth. (**c**) MT. (**d**) EM. (**e**) UAMT. (**f**) CCT. (**g**) CPS. (**h**) URPC. (**i**) Our method

In the BCSS-WSSS, red, green, blue, purple and white indicate tumor, stroma, lymphocyte infiltration areas, necrosis, and other, respectively. For more intuitive visualization, the ground-truth annotations and prediction results are overlaid on the original images, with the corresponding labels displayed in the upper-left corner of each blended image in Fig. 6. In the first two rows of Fig. 6, the tissue boundaries are particularly well-define. Most of the comparative methods are able to roughly delineate the tissue regions; however, their limitations become evident at the boundaries, where segmentation predictions are less precise. In contrast, our proposed method achieves considerably more segmentation predictions, producing smoother and more well-defined boundaries. In the last three rows of Fig. 6, tissue structures are more complex and intertwined, which is challenging to the segmentation task. The proposed method demonstrates higher visual agreement with the ground truth and effectively preserves contour details. These observations indicate that our approach achieves superior segmentation performance, particularly in cases involving intricate cancer tissue structures.

### Ablation study

#### The effectiveness of multi-scale feature aggregation

To evaluate the contributions of the key components of the proposed method, we conducted ablation studies on the 1st HSUMC dataset. The results are summarized in Table 4. In these experiments, M_0_ serves as the baseline network. The M_1_ model, based primarily on the URPC method [26], applies hierarchical consistency (HC) at four decoder levels (S = 4) but does not include the multi-scale convolution module (MSC) or the coarse-fine grained consistency (CFGC). In contrast, M_2_, M_3_, and the proposed method incorporate HC, MSC, and CFGC, with HC applied at two levels (S = 2) in M_2_, three levels (S = 3) in M_3_, and all four levels (S = 4) in the proposed method. Compared with M_0_, M_1_ achieves improvements of 0.92% and 0.70% in IoU and DSC, respectively, demonstrating the effectiveness of hierarchical consistency in encouraging the network to learn consistent feature representations across different decoder levels. To further explore the influence of the number of hierarchical levels, we compared model performance for S ranging from 2 to 4. The proposed method consistently achieves the best results, indicating that leveraging multi-level segmentation outputs provides richer semantic information and enhances segmentation accuracy. Moreover, the addition of MSC and CFGC further improves performance. Compared with M_1_, the proposed method shows steady gains, highlighting the effectiveness of MSC in multi-scale feature aggregation and the benefits of CFGC for guiding semi-supervised learning in histopathology image segmentation.

**Table 4:** Ablation studies on the 1st HSUMC dataset

Method	HC	MSC	CFGC	IoU	DSC
M_0_	w/o	w/o	w/o	0.7335	0.8415
M_1(S=4)_	w/	w/o	w/o	0.7427	0.8485
M_2(S=2)_	w/	w/	w/	0.7463	0.8509
M_3(S=3)_	w/	w/	w/	0.7488	0.8528
The proposed method_(S=4)_	w/	w/	w/	**0.7497**	**0.8536**

#### The effectiveness of different component modules

To evaluate the effectiveness of each component, we conduct ablation experiments on the Camelyon17 dataset. Table 5 reports the quantitative results of different module combinations.

The baseline model with only HC regularization achieves a DSC of 0.9226. Replacing HC with CFGC improves the DSC to 0.9282, indicating that CFGC regularization provides more effective supervision when leveraging unlabeled data. When MSC is introduced together with CFGC, the DSC further increases to 0.9309, demonstrating the benefit of enriching decoder features with multi-scale representations. When HC and CFGC are jointly applied without MSC, the DSC reaches 0.9266, which is lower than using CFGC alone. In contrast, incorporating MSC into the full framework (HC + CFGC + MSC) raises the DSC to 0.9339, achieving the best performance among all configurations. These findings demonstrate that although HC and CFGC each provide measurable gains, MSC serves as a key feature-enhancement component that facilitates more effective interaction between the two consistency constraints, leading to superior segmentation performance.

**Table 5:** Ablation studies of different component modules

Module	HC	CFGC	MSC	DSC
Base	√			0.9226
Base		√		0.9282
Base		√	√	0.9309
Base	√	√		0.9266
Base	√	√	√	**0.9339**

#### The ablation study on the parameterβ

We conducted a sensitivity analysis on the parameter β by setting it to 0.25, 0.5, and 0.75, respectively. As shown in Table 6, the performance remains relatively stable across different β values, with a maximum variation of only 0.37% in DSC. This indicates that the proposed dual-consistency framework is not overly sensitive to the choice of β. Among the tested values, β = 0.5 achieves the best overall performance, suggesting that assigning equal importance to HC and CFGC provides a balanced regularization effect.

**Table 6:** Ablation studies of the parameterβ

β	DSC
0.25	93.02
0.5	**93.39**
0.75	93.24

### Annotation prediction

To further demonstrate the model’s capability in assisting histopathology specialists with making decision on WSIs annotations. We also conducted experiments on WSIs from the 1st HSUMC dataset without annotation. Using the proposed method, predictions were first generated for each image patch and then stitched together to produce the WSI-level segmentation. The contours of the segmented regions were subsequently extracted, with points sampled at 15-pixel intervals. To ensure alignment with the original WSI, the coordinates were scaled according to the magnification factor applied during preprocessing. All sampled points were then compiled into an XML annotation file compatible with the ASAP histopathology image viewer. This enables pathologists to review the automatically generated annotations, evaluate their accuracy, and make any necessary corrections, facilitating the use of these annotations in future studies.

**Fig. 7:**
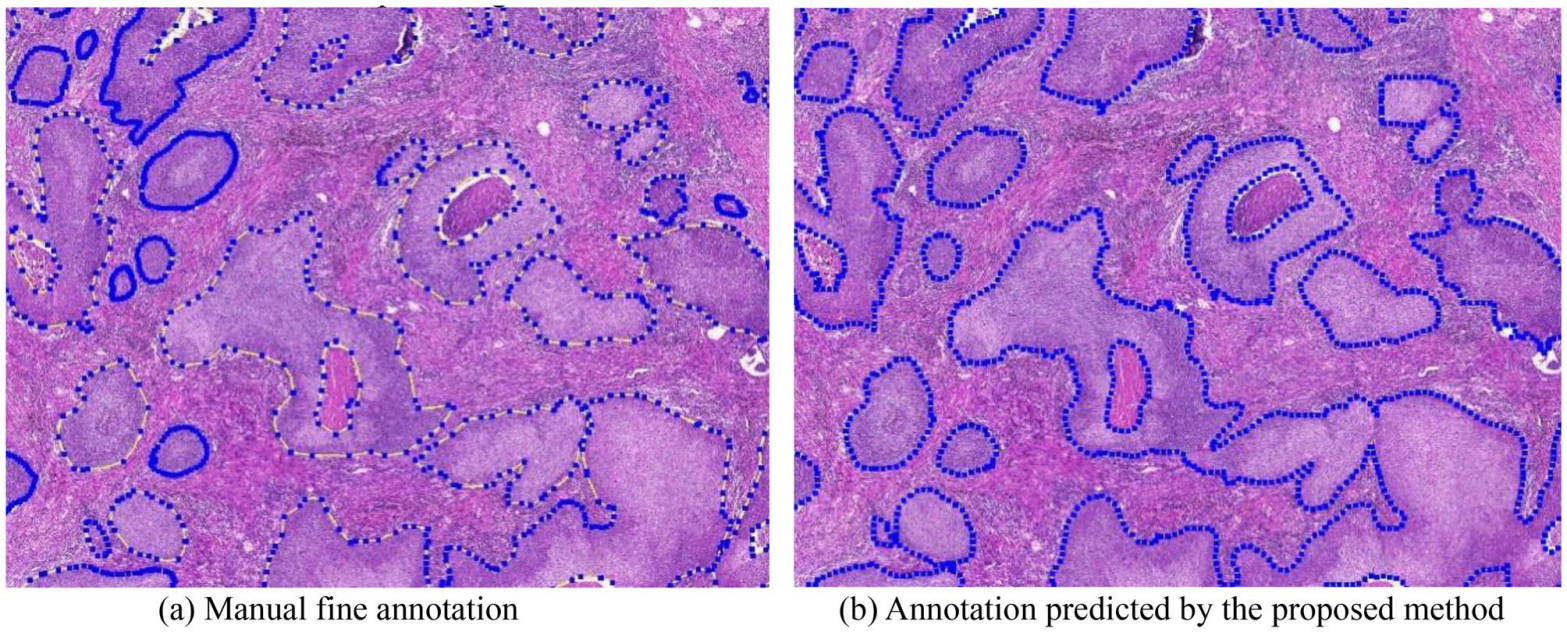
Comparison of manual annotation and the prediction annotation of the proposed method. (**a**) Manual fine annotation, (**b**) Annotation predicted by the proposed method

**Fig. 8:**
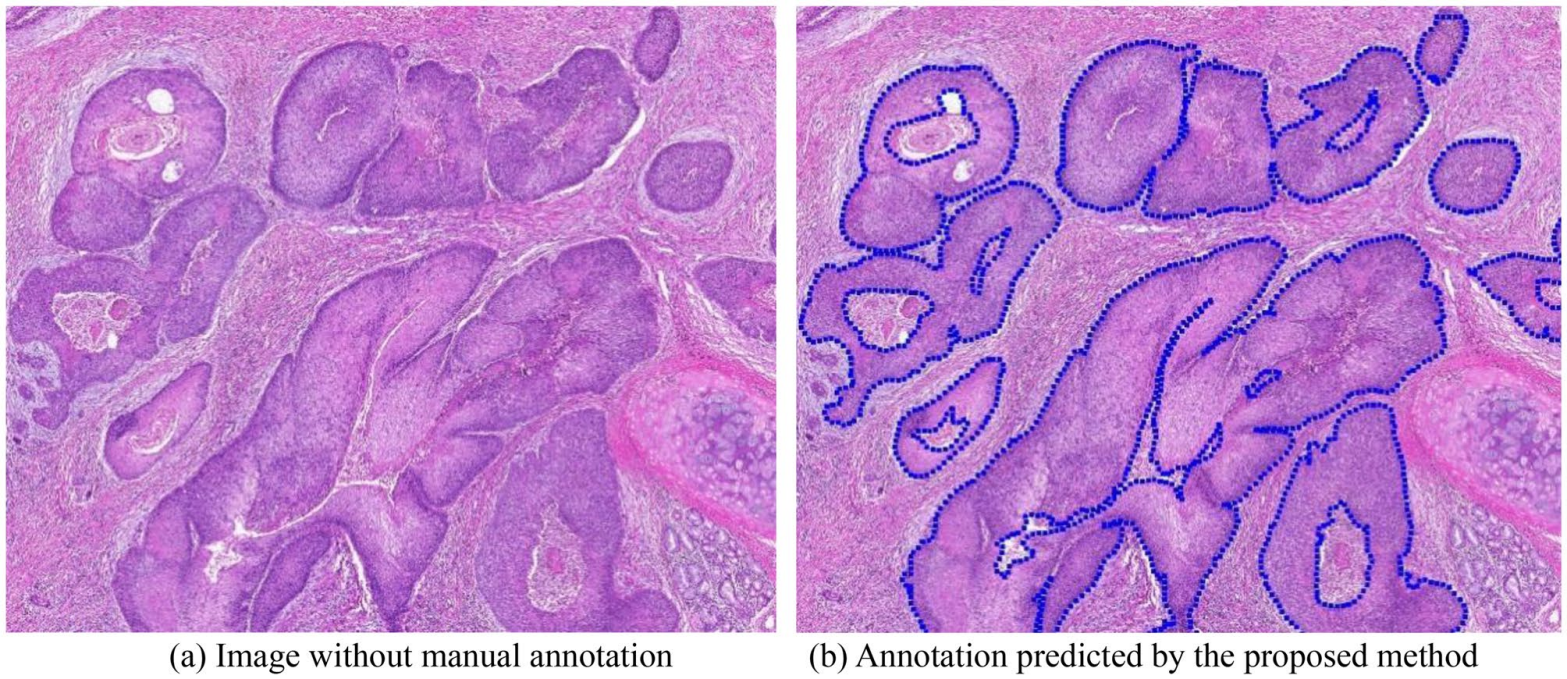
Comparison of images without manual annotation and the prediction annotation of the proposed method. (**a**) Image without manual annotation, (**b**) Annotation predicted by the proposed method

Figure 7 presents a comparison between the annotations generated by the proposed method and the manually curated fine annotations, with cancer regions indicated by blue outlines. Figure 8 shows the segmentation results of a WSI without existing annotations, overlaid on the original slide image. The results demonstrate that the proposed method produces annotations comparable to manual fine annotations. Notably, it achieves accurate and smooth delineation of cancer boundaries at the WSI level, highlighting its effectiveness for large-scale histopathology image segmentation. Furthermore, the method has strong potential to assist in generating XML annotations, thereby substantially reducing the annotation workload for pathologists.

## Conclusion

In this study, we propose a dual-consistency semi-supervised learning framework for histopathology image segmentation. The method integrates two complementary consistency regularizations: hierarchical consistency (HC) and coarse-fine grained consistency (CFGC). Specifically, HC enforces consistency across hierarchical decoder predictions, promoting more stable and coherent feature representations, while CFGC encourages agreement between coarse- and fine-grained segmentation outputs. In addition, a multi-scale convolution module is incorporated to capture rich contextual information, facilitating the recovery of fine structural details. Furthermore, we developed a supplementary annotation technique for whole-slide images (WSIs), which automatically generates XML-based contour annotations. This tool enables clinicians to accurately delineate tumor regions, improving both annotation efficiency and diagnostic workflow. Experimental results on both the self-created 1st HSUMC dataset and public datasets demonstrate the effectiveness of the proposed method, highlighting its potential to support pathologists in clinical diagnosis.

In future work, we plan to further explore the application of our method to assist pathologists in diagnosis. Specifically, we aim to extend the framework to multi-modal studies by integrating hematoxylin and eosin (HE) images with immunohistochemistry (IHC) histopathology images, with the goal of providing more comprehensive support for accurate clinical decision-making.

## Data Availability

The Camelyon17 and BCSS-WSSS dataset used in this study is publicly dataset. The self-collected 1 st HSUMC dataset is not publicly available due to patient privacy concerns but can be obtained from the corresponding author upon reasonable request.
